# A high-resolution physical map integrating an anchored chromosome with the BAC physical maps of wheat chromosome 6B

**DOI:** 10.1186/s12864-015-1803-y

**Published:** 2015-08-12

**Authors:** Fuminori Kobayashi, Jianzhong Wu, Hiroyuki Kanamori, Tsuyoshi Tanaka, Satoshi Katagiri, Wataru Karasawa, Satoko Kaneko, Shota Watanabe, Toyotaka Sakaguchi, Yumiko Hanawa, Hiroko Fujisawa, Kanako Kurita, Chikako Abe, Julio C. M. Iehisa, Ryoko Ohno, Jan Šafář, Hana Šimková, Yoshiyuki Mukai, Masao Hamada, Mika Saito, Goro Ishikawa, Yuichi Katayose, Takashi R. Endo, Shigeo Takumi, Toshiki Nakamura, Kazuhiro Sato, Yasunari Ogihara, Katsuyuki Hayakawa, Jaroslav Doležel, Shuhei Nasuda, Takashi Matsumoto, Hirokazu Handa

**Affiliations:** Plant Genome Research Unit, National Institute of Agrobiological Sciences, Tsukuba, 305-8602 Japan; Advanced Genomics Laboratory, National Institute of Agrobiological Sciences, Tsukuba, 305-8602 Japan; Bioinformatics Research Unit, National Institute of Agrobiological Sciences, Tsukuba, 305-8602 Japan; Laboratory of Plant Genetics, Graduate School of Agriculture, Kyoto University, Kyoto, 606-8502 Japan; Cereal Science Research Center of Tsukuba, Nisshin Flour Milling Inc., Tsukuba, 300-2611 Japan; Laboratory of Plant Genetics, Graduate School of Agricultural Science, Kobe University, Kobe, 657-8501 Japan; Core Research Division, Organization of Advanced Science and Technology, Kobe University, Kobe, 657-8501 Japan; Institute of Experimental Botany, Centre of the Region Haná for Biotechnological and Agricultural Research, CZ-78371 Olomouc, Czech Republic; Wheat Breeding Group, NARO Tohoku Agricultural Research Center, Morioka, 020-0198 Japan; Institute of Plant Science and Resources, Okayama University, Kurashiki, 710-0046 Japan; Kihara Institute for Biological Research, Yokohama City University, Yokohama, 244-0813 Japan

**Keywords:** Centromere, Chromosomal rearrangement, Chromosome 6B, DNA marker, Gene order, Nucleolus organizer region, BAC physical map, RH map, Synteny, Wheat

## Abstract

**Background:**

A complete genome sequence is an essential tool for the genetic improvement of wheat. Because the wheat genome is large, highly repetitive and complex due to its allohexaploid nature, the International Wheat Genome Sequencing Consortium (IWGSC) chose a strategy that involves constructing bacterial artificial chromosome (BAC)-based physical maps of individual chromosomes and performing BAC-by-BAC sequencing. Here, we report the construction of a physical map of chromosome 6B with the goal of revealing the structural features of the third largest chromosome in wheat.

**Results:**

We assembled 689 informative BAC contigs (hereafter reffered to as contigs) representing 91 % of the entire physical length of wheat chromosome 6B. The contigs were integrated into a radiation hybrid (RH) map of chromosome 6B, with one linkage group consisting of 448 loci with 653 markers. The order and direction of 480 contigs, corresponding to 87 % of the total length of 6B, were determined. We also characterized the contigs that contained a part of the nucleolus organizer region or centromere based on their positions on the RH map and the assembled BAC clone sequences. Analysis of the virtual gene order along 6B using the information collected for the integrated map revealed the presence of several chromosomal rearrangements, indicating evolutionary events that occurred on chromosome 6B.

**Conclusions:**

We constructed a reliable physical map of chromosome 6B, enabling us to analyze its genomic structure and evolutionary progression. More importantly, the physical map should provide a high-quality and map-based reference sequence that will serve as a resource for wheat chromosome 6B.

**Electronic supplementary material:**

The online version of this article (doi:10.1186/s12864-015-1803-y) contains supplementary material, which is available to authorized users.

## Background

Common wheat (*Triticum aestivum* L.) is an allohexaploid species with three distinct genomes, A, B and D, which have been defined by genome analysis [1]. The wheat genome consists of 21 chromosomes, and each of the three subgenomes contributes 7 chromosomes [2, 3]. The genome has a total length of 16.9 Gb [4], which is extremely large compared to the sequenced genomes of other grass species: rice (389 Mb; [5]), *Brachypodium distachyon* (272 Mb; [6]), sorghum (730 Mb; [7]), maize (2.3 Gb; [8]) and barley (5.1 Gb; [9]). Most of the wheat genome is occupied by various types of repetitive DNA sequences, which account for more than 80 % of the entire genome [10]. Although wheat has been widely used in cytogenetics because of the high visibility of its chromosomes, complex features, such as its large size, high repeat content and polyploidy, have hampered molecular approaches to date, particularly whole-genome sequencing. However, recent technological advances have paved the way for genomic approaches that work for even complex genomes, such as wheat. For example, whole-genome sequences of the common wheat cultivar ‘Chinese Spring’ (CS), wild diploid wheat *Triticum urartu* (A genome progenitor of common wheat) and wild diploid goat grass *Aegilops tauschii* (D genome progenitor of common wheat) were analyzed using next-generation sequencing (NGS) technology [11–13].

The International Wheat Genome Sequencing Consortium (IWGSC) has been coordinating wheat genome sequencing to enhance knowledge of the structure and function of the bread wheat genome (http://www.wheatgenome.org/) and to provide tools to facilitate the breeding of improved varieties. The consortium has adapted a strategy that relies on the purification of individual chromosome arms from wheat telosomic lines [2] by flow-cytometric sorting to reduce the sample complexity [14] and obtain chromosome-specific genomic data. Recently, the IWGSC presented a draft sequence of all 21 wheat chromosomes [15] in which the DNA of flow-sorted fractions was sequenced by NGS. To obtain a reference genome sequence using BAC-by-BAC sequencing, physical mapping of the BAC contigs (hereafter reffered to as contigs) is currently underway for each of the 21 chromosomes [4]. To date, BAC-based physical maps have been developed for chromosomes 1A, 1B, 3B and 6A [16–21]. Most recently, during the preparation of this paper, two additional physical maps for chromosome arms 3DS and 5DS were released [22, 23].

Our group is responsible for sequencing wheat chromosome 6B (http://komugigsp.dna.affrc.go.jp/index.html) under the framework of IWGSC. This chromosome is the third largest chromosome in common wheat, with an estimated molecular size of 914 Mb: 415 Mb representing the short arm (6BS) and 498 Mb representing the long arm (6BL) [4]. As a first step, we conducted whole-chromosome shotgun sequencing using DNA that had been amplified from the flow-sorted chromosome arms 6BS and 6BL with massively parallel 454 pyrosequencing [24]. The survey sequence data were highly informative for determining the genomic composition of chromosome 6B. Within a total of 508 Mb of assembled sequence (56 % of the size of 6B), 4798 gene loci were predicted, and more than 70 % of the 6B assembly consisted of repetitive sequences. Functional non-protein-coding RNAs, such as micro-RNA, transfer RNA and ribosomal RNA (rRNA), were also identified. In particular, the short arm (6BS) is characterized by the presence of secondary constriction, the nucleolus organizer region (NOR), which features a locus for the rRNA genes (rDNA locus) [25, 26]. The NOR is designated as *Nor-B2* [27]. We identified several contigs containing the rDNA locus, and they exhibited extremely high read depths, indicating that these contigs were part of the NOR on 6BS.

In this study, we established a BAC-based physical map of chromosome 6B with the goal of developing a high-quality reference sequence for the wheat genome. To achieve this goal, two BAC libraries were constructed using arm-specific DNA samples. In addition, we used two recently developed genomic tools to physically map all of the contigs onto chromosome 6B. This process was performed using insertion site-based polymorphism (ISBP) markers (Kaneko et al. in preparation) that were designed from the junction sequences between transposable elements and their flanking sequences [28], and a radiation hybrid (RH) mapping panel (Watanabe et al. in preparation) that was derived from a series of chromosome deletion lines generated by γ-ray irradiation [29]. Combining the above genomic resources with other wheat-specific data, we were able to develop a robust system to anchor and order contigs onto the high-resolution RH map of chromosome 6B. The physical map not only allows us to understand key structural features and the evolutionary history of chromosome 6B but will also enable the use of genome sequencing to create a high-quality map-based reference sequence in the near future.

## Results and discussion

### Construction of 6B arm-specific BAC libraries

Chromosome arms 6BS and 6BL were purified by flow-cytometric sorting from the double ditelosomic line 6B of CS [30], as described by Tanaka et al. [24]. The average purities in the sorted fractions were 85 and 92 % for 6BS and 6BL, respectively. High-molecular-weight DNA prepared from the purified arms was used to construct the BAC libraries TaaCsp6BShA and TaaCsp6BLhA, which were specific to 6BS and 6BL, respectively (http://olomouc.ueb.cas.cz/dna-libraries/cereals). The libraries consisted of 57,600 and 76,032 BAC clones with average insert lengths of 132 and 130 kb, representing 15.3 and 18 genome equivalents of the estimated size of 6BS and 6BL, respectively (Table 1).

**Table 1:** Arm-specific BAC libraries on chromosome 6B

	6BS	6BL
Library code	TaaCsp6BShA	TaaCsp6BLhA
Number of clones	57,600	76,032
Average insert size	132 kb	130 kb
Coverage	15.3x	18.0x
Purity of the sorted fraction	85 %	92 %

### Construction of a BAC-based physical map of wheat chromosome 6B

**Fig. 1:**
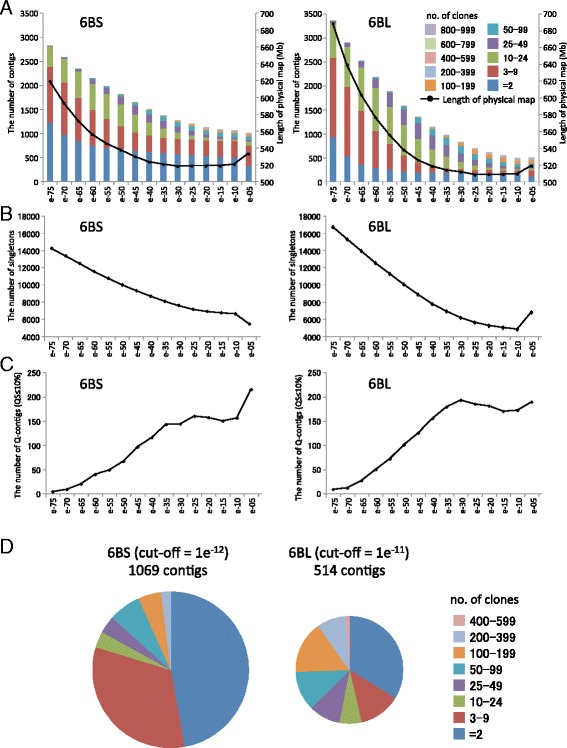
Comparison of the contig assemblies between cut-offs of 1e^−75^ and 1e^−05^ in the chromosome arms of 6BS and 6BL. **a** The number of contigs and their cumulative sizes at each cutoff value. The colors in the bars represent the number of clones comprising one BAC contig. **b** Total number of singleton clones in the physical map at each cut-off value. **c** Total number of Q-contigs containing ≤10 % questionable clones in the physical map at each cut-off value. **d** Total number of contigs in the physical maps of 6BS and 6BL with an optimal cut-off value. Eight colors in circles represent the number of clones comprising one BAC contig. The sizes of the circles indicate the relative total number of contigs in the physical map of 6BS and 6BL

Whole Genome Profiling (WGP™) [31] was used to fingerprint a total of 41,472 and 49,920 BACs from 6BS and 6BL, representing 13 times the total physical lengths of the arms, respectively (Additional file 1). After deconvolution for the assignment of high-quality reads, we made several modifications to the standard WGP procedure following Philippe et al. [32] to ensure a highly accurate physical map. We initially excluded BACs with less than 30 % (<7 tags) or more than 2.5 times (>51 tags) the average number of tags per BAC from the analysis based on the average number of tags per BAC (20.6 and 21.8 for 6BS and 6BL, respectively) (Additional files 1 and 2). This filtering resulted in the availability of 28,828 and 38,953 BACs for assembly on 6BS and 6BL, representing 9.2 and 10.2 times the total physical length, respectively (Additional file 3). Assembly of the fingerprints of 6BS and 6BL was performed using FingerPrinted Contigs (FPC) software [33]. A stepwise method was used to improve the quality of the contigs in the assembly, which was initially defined during the physical map construction of chromosome 3B [20] and then modified for the WGP fingerprints [32]. Using the 6B-specific parameters at final cut-off values of 1e^−12^ and 1e^−11^, which were determined based on the balance among the number of contigs, the number of Q-contigs and the coverage of the physical map, we constructed the first physical maps of 6BS and 6BL with 1069 and 514 contigs, respectively (Additional file 3 and Fig. 1; the URGI site of the wheat physical map viewer, https://urgi.versailles.inra.fr/gb2/gbrowse/wheat_phys_pub/).

These physical maps still contained a large number of small contigs (2 BACs/contig), particularly on 6BS (Fig. 1d). However, the small contigs might be redundant because of their low-quality fingerprints containing genomic fragments that were already present in the large contigs. In addition, they had the potential to carry BAC clones derived from chromosomes other than 6B due to DNA contamination during the chromosome-sorting process (the purity of the sorted fractions was approximately 85–92 %; Table 1). To avoid any chromosomal redundancy and contamination, we eliminated 765 and 129 of these small and low-confidence contigs from the above physical map constructed primarily on the two arms of 6BS and 6BL, respectively, based on the results of a homology search of the WGP tag sequences against the whole-genome survey sequence [15] and/or the depth of the BAC clones within each contig. Finally, we constructed the BAC-based physical map of chromosome arms 6BS and 6BL, which contained 304 and 385 contigs, respectively (Table 2).

**Table 2:** Physical maps of chromosome arms 6BS and 6BL

	6BS	6BL
Number of BACs in the contigs	20,225	33,830
Number of contigs	304	385
Estimated size (chromosome coverage^a^)	359 Mb (87 %)	475 Mb (95 %)
Contigs N50 (kb)	2302	2508
Contig L50	52	61
Number of MTP BACs	3076	4557

^a^Coverage was calculated based on the estimated size of each arm: 415 Mb for 6BS and 498 Mb for 6BL

The above process greatly improved our BAC-based physical map. The first physical maps revealed estimated sizes of 492 and 495 Mb that corresponded to 119 % and 99 % of the arm lengths, N50 values of 1503 and 2422 kb and L50 values of 87 and 65 contigs for 6BS and 6BL, respectively (Additional file 4). However, the final maps showed remarkable increases in the N50 values (from 1503 to 2302 kb for 6BS and from 2422 to 2508 kb for 6BL) and a decrease in the L50 values (from 87 to 52 for 6BS and from 65 to 61 for 6BL), although the total lengths for both arms decreased to 359 and 475 Mb, covering approximately 87 and 95 % of the entire genomic region for 6BS and 6BL, respectively (Additional file 4; Table 2). The reason for the lower coverage of 6BS remains unclear, but it is likely a consequence of the purity of the sorted chromosome arm DNA (Table 1), the number of WGP tags on each BAC (Additional file 2) or differences in the genomic structure between 6BS and 6BL (e.g., the *Nor-B2* region with highly repetitive rRNA gene sequences).

Further overlap analysis of neighboring clones within each contig using the FPC MTP module allowed us to select 3076 and 4557 minimal tiling path (MTP) clones along the two arms, laying a foundation for the map-based genomic sequencing of the entire chromosome 6B (Table 2).

### Anchoring of chromosome 6B-specific markers to determine the physical location of the BAC contigs

To obtain a pseudomolecule sequence, which is the final goal of IWGSC, it is essential to map the contigs onto their specific genomic regions on wheat chromosome 6B. For that purpose, a set of anchors is needed. As shown in Table 3, we first searched through the available marker information in public databases, such as the “GrainGenes CMap” (http://wheat.pw.usda.gov/cmap/) and the “National BioResource Project (NBRP)-Wheat, Japan” (http://www.shigen.nig.ac.jp/wheat/komugi/), and 208 SSR and RFLP markers (already converted to STS markers) were found to be potentially useful for chromosome 6B. Second, we collected a resource of 751 DNA markers from the PCR-based Landmark Unique Gene (PLUG) Database (http://plug.dna.affrc.go.jp/). These markers are known EST-PCR markers that were developed by taking advantage of the orthologous gene conservation between rice and wheat [34, 35]. To develop new markers, we designed 390 and 1829 PCR primer pairs from the genic sequences by using the barley GenomeZipper (chromosome 6H) [36] and the syntenic relationships of rice (chromosome 2), *B. distachyon* (chromosome 3) and sorghum (chromosome 4), respectively. Finally, using the survey sequences of wheat chromosome 6B [15], we prepared an additional 2000 ISBP PCR primer pairs (1000 for each chromosome arm) from the junction sequences between transposable elements and their flanking sequences ([28], Kaneko et al. in preparation).

**Table 3:** Molecular markers used for the physical mapping

Marker category	Developed	Anchored to BAC contig	On integrated physical map
		(success rate)	
Genic markers^a^	2970	1164 (39.2 %)	1159
SSR and STS-RFLP markers	208	127 (61.1 %)	127
ISBP marker	2000	1293 (64.7 %)	1290
Total	5178	2584 (50.0 %)	2576

^a^Genic markers include the PLUG marker, GenomeZipper-based markers and orthologous sequence markers

**Fig. 2:**
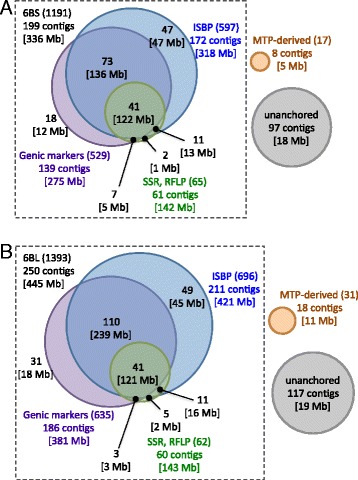
Relative contributions of different types of molecular marker used for anchoring by PCR screening for the physical maps of the chromosome arms of 6BS (**a**) and 6BL (**b**). The number of markers anchored on the BAC contigs is indicated in parentheses after the marker type. The number of contigs anchored and their physical size (in brackets) are indicated for each marker type. The Venn diagram in the square illustrates the relative contribution of each marker type to the number and total size of contigs anchored by one or more marker types in the PCR screening of BAC libraries. Single circles in orange or gray represent the number and total size of the newly anchored contigs with MTP-derived markers or unanchored contigs, respectively, which were not anchored by the PCR screening

After validating the quality and effectiveness of the above 5178 DNA markers by PCR, we found that up to 2584 primer pairs (1191 for 6BS and 1393 for 6BL) could amplify a single sharp band from the DNA samples of flow-sorted chromosome 6B arms or pooled arm-specific BAC libraries. These primer pairs represent good markers of the chromosomal locations of the contigs (Table 3). As shown in Fig. 2, using large-scale PCR-screening of two arm-specific BAC libraries, we successfully localized the above 2584 anchor markers onto individual BAC clones among a total of 449 contigs (199 on 6BS and 250 on 6BL, 5.8 markers per contig). The ISBP markers provided more effective anchoring of the contigs compared to the genic markers (89 % versus 77–80 %), supporting previous results ([20]; Kaneko et al. in preparation). In addition, 271 markers were specific to wheat chromosome 6B and were polymorphic between the two wheat varieties CS and ‘Mironovskaya 808’ (M808), which were the parents used to generate the mapping population of recombinant inbred lines (RILs) [37] that was used for genetic mapping of contigs.

To anchor the contigs without any of 2584 6B-specific anchor marker hits, we developed a point-by-point supplemental set of 284 novel 6B-specific markers (92 converted from the markers specific to wheat group-6 chromosomes using the survey sequence data [15] and 192 from the genomic sequences of the MTP BAC clones in the corresponding contigs). Finally, the total number of contigs anchored by the chromosome 6B markers was increased to 475, including 207 contigs anchored by 1324 markers on 6BS and 268 contigs anchored by 1544 markers on 6BL (Table 4).

**Table 4:** Characteristics of the BAC contigs in the physical map of chromosome 6B

	6BS (size, coverage^a^)	6BL (size, coverage^a^)	6B (size, coverage^a^)
BAC contigs on the RH map	201 (340.2 Mb, 82.0 %)	261 (454.5 Mb, 91.3 %)	462 (794.8 Mb, 87.0 %)
BAC contigs not on the RH map but anchored with markers	6 (1.05 Mb, 0.25 %)	7 (1.12 Mb, 0.22 %)	13 (2.17 Mb, 0.24 %)
BAC contigs not anchored with markers	97 (17.8 Mb, 4.3 %)	117 (19.0 Mb, 3.8 %)	214 (36.8 Mb, 4.0 %)

^a^Coverage was calculated based on the estimated size of each arm and the entire chromosome: 415 Mb for 6BS; 498 Mb for 6BL; and 914 Mb for 6B

**Fig. 3:**
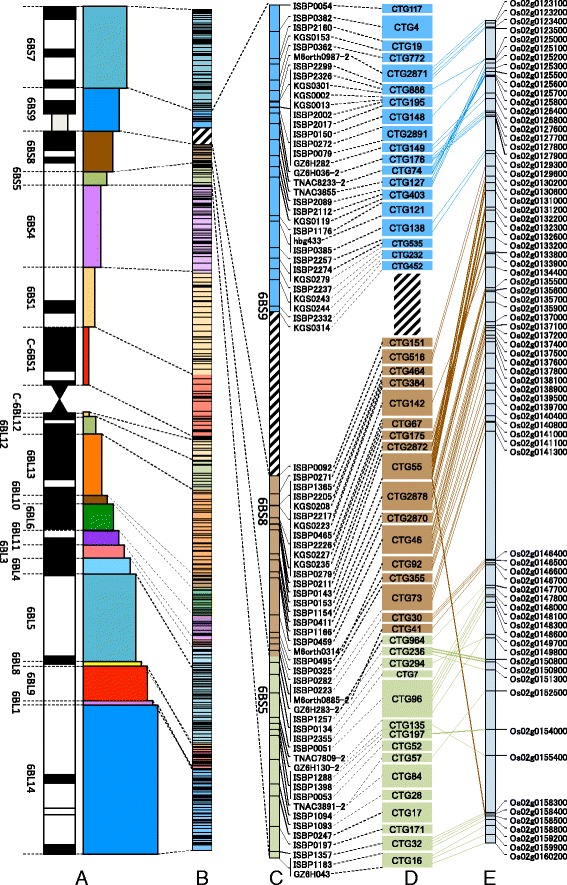
RH map of chromosome 6B and contig order in three deletion bins of 6BS. **a** Deletion bin map of chromosome 6B. Twenty deletion bins are illustrated by colored boxes. **b** RH map of chromosome 6B. The map is divided into segments corresponding to deletion bins with the same colors. The striped segment on 6BS represents the putative nucleolus organizer region (*Nor-B2*). The segment colored in black represents a boundary between 6BS and 6BL. **c** RH map at deletion bins 6BS9, 6BS8 and 6BS5. The marker name is indicated to the right of the chromosome. **d** BAC contigs assigned to the RH map. The name of the BAC contigs is displayed in colored boxes, the size of which reflects the relative contig size. The colors of the boxes correspond to those of the deletion bins. **e** Corresponding region of rice chromosome 2. Each dashed line represents the relationship between a genic marker located in a wheat BAC contig and the orthologous rice gene

First, we attempted the genetic mapping of contigs on chromosome 6B. The use of 226 genetic markers located on the genetic map generated with the RIL population mentioned above led to the chromosomal assignment of 118 contigs (58 on 6BS and 60 on 6BL), encompassing a genomic region of 287 Mb, on the two arms, which represents 31 % of chromosome 6B (data not shown). However, genetic markers alone were not sufficient for the physical mapping of all contigs that were assembled for wheat chromosome 6B. To overcome this problem, we used RH mapping in chromosome deletion lines that were produced by γ-ray irradiation to determine the chromosomal locations and orders of all of the anchor DNA markers for the 6B contigs. This strategy was used because RH mapping provides results that are independent of polymorphisms. Thus, it is a powerful tool for the high-resolution mapping of wheat chromosomes [29, 38]. Using the RH panel of 355 lines and 21 chromosome 6B deletion lines made by Endo and Gill [39], we established an RH map with one linkage group consisting of 653 markers (448 loci), in which a total of 1277 obligate breaks were identified with an estimated resolution of approximately 622 kb/break (Table 5; Fig. 3b). The total length of the RH map was 1560.7 cR, indicating an average space of 3.5 cR/loci and an average distance of 509 kb/cR (the physical length used herein was estimated from the cumulative size of the contigs). Based on the RH mapping results, 462 FPC-assembled contigs were mapped. However, 18 of these mapped contigs (4 on 6BS and 14 on 6BL) were judged to be chimeric because the markers anchored to the contig were located on distant, unrelated positions of the RH map. Therefore, we split each of these 18 contigs into two distinct contigs based on the FPC data and the anchored marker positions. Finally, we determined the locations and/or directions of 480 contigs on wheat chromosome 6B, including the contigs that mapped to the same locus (Additional file 5). These contigs revealed a total physical length of 794.8 Mb (340.2 and 454.5 Mb, respectively, on 6BS and 6BL), representing approximately 87 % of chromosome 6B (Table 4). Although 227 contigs, one third of the total contigs, still remained unmapped, most of these contigs were assembled using a small number of BAC clones with a total physical length of 39.0 Mb and an average size of 172 kb per contig (maximum = 386 kb, minimum = 81 kb), so they cover only 4.2 % of chromosome 6B.

**Table 5:** Integrated physical map and map resolution

Deletion bin	Markers	Loci	Length (cR)	Mapped contigs	Mapped contig size (Mb)	Obligate breaks^a^	Map resolution (Mb)
6BS7	91	64	174.6	64	84.3	180	0.47
6BS9	34	22	83.8	19	26.8	57	0.47
6BS8	25	15	33.1	17	33.0	29	1.14
6BS5	18	13	35.8	15	26.4	32	0.83
6BS4	54	43	145.4	39	76.9	115	0.67
6BS1	35	30	209.1	29	61.5	134	0.46
C-6BS1	34	21	102.7	22	31.5	58	0.54
C-6BL12	13	10	42.2	10	7.6	19	0.40
6BL12	15	11	50.8	11	17.1	27	0.63
6BL13	39	33	154.3	34	60.3	103	0.59
6BL10	9	8	25.4	6	7.3	16	0.46
6BL6	18	16	48.8	15	28.6	36	0.79
6BL11	17	9	41.7	14	18.1	27	0.67
6BL3	9	7	21.1	8	21.4	13	1.65
6BL4	11	11	34.5	9	10.5	25	0.42
6BL5	69	50	155.2	51	87.6	128	0.68
6BL8	3	2	5.8	1	3.6	2	1.80
6BL9	29	19	43.9	25	35.0	36	0.97
6BL1	2	2	3.2	1	1.7	2	0.84
6BL14	128	62	149.3	90	155.7	144	1.08
6B	653	448	1560.7	480	794.8	1277	0.62

^a^The number of obligate breaks in each deletion bin was calculated by using the map region between the distal and proximal markers

Our RH mapping could offer more refined deletion bins for chromosome 6B than were previously available [40]. The 18 breakpoints in the 6B deletion lines and 653 markers defined 20 chromosomal blocks (7 on 6BS and 13 on 6BL). These 20 blocks were designated as improved deletion bins (Fig. 3a). Each deletion bin was denoted by the name of the deletion line and the breakpoint at the proximal end of the bin (Fig. 3a). Based on the number of obligate breaks and the bin size (calculated from the cumulative size of the contigs), the resolution of all 20 deletion bins was estimated to range from 0.4 to 1.8 Mb/break (Table 5). Five bins (6BS8, 6BL3, 6BL8, 6BL9 and 6BL14) showed a relatively low resolution with >900 kb/break, which may have occurred for the following reasons: 1) the large cumulative size of the contigs with a limited number of obligate breaks (e.g., 6BS8, 6BL3 and 6BL9); 2) one locus mapped by a large number of markers (e.g., 6BL14); and 3) a bin mapped with only one large contig (e.g., 6BL8). Deletion bins that mapped to regions close to the centromere a remarkably high resolution. For example, bin C-6BL12 had the highest resolution, 0.40 Mb/break (Table 5). This result shows that RH mapping is useful for the chromosomal assignment of contigs, even within the centromeric region, where recombination events are extremely rare.

Notably, the largest space between markers on the RH map was located between KGS0314 (6BS9) and ISBP0092 (6BS8), which were separated by 29.2 cR (Fig. 3c). Clearly, this chromosomal site was associated with the chromosome breakpoint of deletion line 6BS-9, which was located near *Nor-B2* [39]. These results suggest that this space over 29.2cR corresponds to the NOR region (see the following section).

Although 350 of the 480 contigs (152 on 6BS and 198 on 6BL) were specifically positioned in order along chromosome 6B (Additional file 5), 130 contigs were localized within a total of 47 loci (16 on 6BS and 31 on 6BL), which indicated that multiple contigs resided within the same locus. For example, 4 of the 13 loci within the bin of 6BL14 carried 5 to 7 contigs in which the relative order was unknown at this stage. However, 80 of these 130 contigs (21 on 6BS and 59 on 6BL) were anchored by genic markers with orders that were estimated from the syntenic relationships of rice, *B. distachyon* and sorghum. These efforts led to the successful determination of the physical locations of a total of 430 contigs, representing a total length of 761.6 Mb and covering approximately 83 % of chromosome 6B (Additional file 5).

In the present study, we developed an RH map for chromosome 6B and determined the order of the contigs. Our study clearly demonstrates the appropriateness and effectiveness of the RH mapping approach for ordering contigs on wheat chromosome compared to typical strategies, such as genetic mapping and deletion bin mapping. The RH mapping method has important advantages. It produces uniform chromosomal breaks, allows for direct localization and does not require marker polymorphisms. Thus, it allows for the determination of the chromosomal locations and order of contigs with high resolution and accuracy.

RH mapping was able to define the order of 2860 markers based on contig order (Additional file 5). The marker order on the physical RH map provides more detail and a higher resolution compared to previous deletion bin mapping of chromosome 6B [35, 40–43]. Although the marker density of the 6B RH map (3.6 markers/Mb) is lower than those of other physical maps (10.1 and 11 markers/Mb on 1BS and 1BL, respectively; [18, 19]), all of our markers are PCR markers, which will be valuable for future map-based gene cloning and marker-assisted selection in breeding compared to the array-based markers used in the previous studies.

### Fine mapping of the *Nor-B2* region on the short arm of chromosome 6B

The integration of FPC-assembled BAC contigs with a high-resolution RH map of chromosome 6B allowed us to characterize several features that are specific to the genomic composition and structure of chromosome 6B. In common wheat, the majority of the highly repeated rRNA genes for 18S, 5.8S and 25S RNAs (rDNA unit) are located at the NOR loci on chromosome arms 1BS and 6BS, as well as at minor sites on chromosome arms 1AS and 5DS, which have been designated *Nor-B1*, *Nor-B2*, *Nor-A1* and *Nor-D3*, respectively [27]. *Nor-B2* is recognized as the secondary constriction on satellite chromosome 6B, which indicates that genes in *Nor-B2* are transcriptionally active [26].

**Fig. 4:**
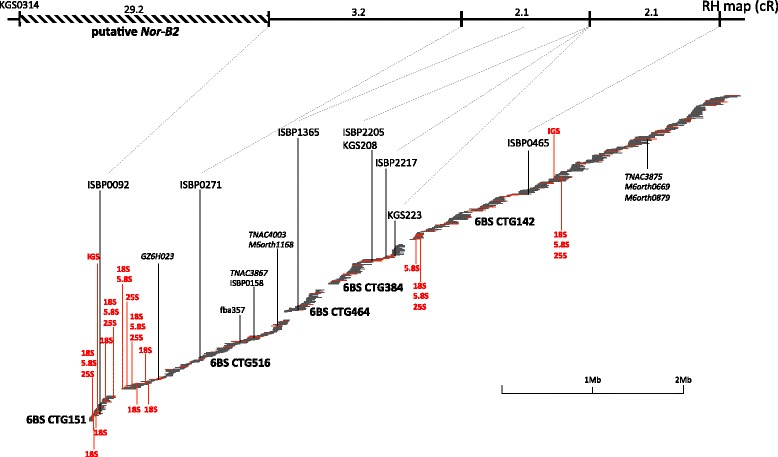
BAC contigs including rRNA gene sequences. The 6BS RH map from a distal locus of KGS0314 on bin 6BS9 to a proximal locus ISBP0465 on bin 6BS8 is shown at the top of the figure. Five BAC contigs corresponding to this region—CTG151, CTG516, CTG464, CTG384 and CTG142—are shown, in which each black line shows the individual BAC clone and the red line indicates the MTP clone. Red vertical lines indicate the positions of three rRNA genes for 18S, 5.8S and 25S and the IGS marker (represented in red characters). The other black vertical lines indicate the positions of the anchoring markers (represented by black characters). Genic markers are shown in italics

Our RH mapping defined a position for *Nor-B2* on chromosome 6BS (Fig. 3b, c). To determine the physical position of *Nor-B2* in detail, we developed one DNA marker using the intergenic spacer sequences (IGSs) between the 18S and 25S rRNA genes [44]. Screening of our BAC libraries with the IGS marker revealed that two contigs, CTG151 (2.6 Mb) and CTG142 (3.7 Mb), located in the deletion bin of 6BS8, putatively contained the rRNA genes (Fig. 3d). Contig CTG151 was mapped onto a genomic region immediately downstream of the estimated *Nor-B2* site using one ISBP marker, ISBP0092, and contig CTG142 had a chromosomal position that was 7.5 cR downstream of that marker on the RH map. Although three other contigs, CTG516 (1.9 Mb), CTG464 (0.4 Mb) and CTG384 (0.8 Mb), were located between CTG151 and CTG142 (Fig. 3d), no MTP clones were detected in these contigs by PCR screening using the IGS marker. However, BLAST analysis of genomic sequences of MTP BAC clones (unpublished data) clearly confirmed the presence of an rDNA unit within the BAC contigs of not only CTG151 and CTG142 but also CTG516 (Fig. 4).

Previous research has identified approximately 5500 rRNA genes on chromosome 6B [45]. Considering that the length of each copy (rDNA unit and adjacent IGS) is approximately 9 kb [46], the complete region encompassing the *Nor-B2* locus could be estimated as approximately 16.5 Mb. Although the five BAC contigs showed a total physical length of 9.4 Mb, the copy numbers of the rRNA genes detected within them were extremely limited (Fig. 4). Therefore, the current BAC-based physical map partially covers the border region (proximal to the centromere) of the *Nor-B2* locus, and it does not include the core rDNA array region of *Nor-B2*. Furthermore, the physically mapped BAC contigs in the genomic region on the other side (proximal to telomere) of the *Nor-B2* locus do not contain the rRNA genes.

Interestingly, all of the rRNA genes discovered on the above contigs that mapped near the *Nor-B2* locus appeared to be interrupted by other types of genomic sequences, including transposons and other genes. This is in striking contrast to the NOR region in rice. In rice (*Oryza sativa* L. ssp. *japonica*), the rRNA genes are distributed in a uniform array throughout the NOR, encompassing a region of approximately 3.5 Mb on chromosome 9 (Additional file 6) [47, 48]. Moreover, the rDNA units have been found to exist as clusters in tandem arrays, even within the border region of the NOR, demonstrating a clear boundary between the rDNA repeat and its flanking region [49]. In the region flanking the rDNAs on rice chromosome 9, Ty3/*gypsy* retrotransposons accumulates at a density twice as high as that of the entire rice genome [5, 49]. Regarding chromosome 6B, two ISBP markers, ISBP0092 and ISBP0465, were designed based on the junction sequences, including the *gypsy*-type retrotransposon (Additional file 6), and these mapped close to the positions of the rRNA genes on CTG151 and CTG142, respectively (Fig. 4). The other five ISBP markers mapped to the same region (Fig. 4), including other types of repeat sequences, such as DNA transposons (CACTA and MITE) and unknown repeat sequences (Additional file 6). We previously found that the DNA transposon diffusion is involved in the propagation of micro RNA genes and specific transfer RNA genes in wheat chromosome 6B based on the analysis of 6B survey sequences [24]. The ambiguity found in the border region of *Nor-B2* might be another example of specific transposons containing rRNA genes becoming diffused around the border region of *Nor-B2*, resulting in insertions of the rRNA genes into the genomic sequence outside of the rDNA array. We identified three gene loci (GZ6H023 and TNAC3867 on CTG516, TNAC3875 on CTG142) that displayed colinearity between wheat and rice (Figs. 3e and 4), and other mapped genes on CTG516 were also found to have conserved gene order relative to rice chromosome 2. These results support the concept that the border region of *Nor-B2* originally possessed a structure similar to that of rice; however, the occurrence of genomic rearrangements due to the transposition of various repeat sequences has resulted in the present ambiguity at the proximal border of *Nor-B2*. This feature may reflect general characteristics of the wheat genome, which contains highly repetitive sequences. A detailed evaluation of the structure of *Nor-B2* and its relationship to the repetitive sequences will be presented in future sequencing studies.

### Fine mapping of the centromere on wheat chromosome 6B

**Fig. 5:**
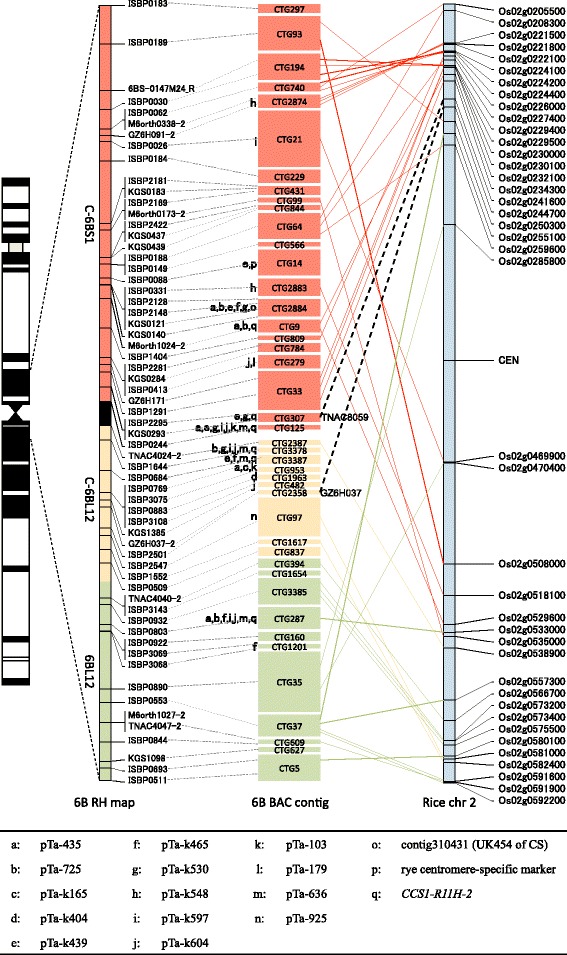
BAC contigs located in the centromeric region of chromosome 6B. The proximal three bins on chromosome 6B, C-6BS-1, C-6BL-12 and 6BL12, are represented in colored segments of the RH map, corresponding to the colors of each bin in Fig. 4a. The black segment represents the boundary between 6BS and 6BL. The marker name is indicated to the right of the map. BAC contigs assigned to the RH map are represented by colored boxes, the sizes of which reflect the relative contig sizes. The names of 17 centromeric sequences used as queries for the BLAST search are listed in the table at the bottom of the figure, and each sequence is represented by a letter from ‘a’ to ‘q’. These letters, shown on the right of the BAC contigs, denote the presence of the corresponding centromeric sequences in those contigs. The corresponding region of rice chromosome 2 is aligned on the right side of the figure. Each colored line represents the relationship between a genic marker located in a wheat BAC contig and the orthologous rice gene. Black dashed lines indicate the relationships between two wheat centromeric genic markers, TNAC8059 on CTG307 (C-6BS1) and GZ6H037 on CTG2358 (C-6BL12), and their orthologous genes in rice

In multicellular eukaryotes, the chromosomal region known as the centromere plays an important role in both mitotic and meiotic nuclear divisions. Centromeres in wheat are associated with highly repetitive Ty3/*gypsy* elements. Moreover, the regions surrounding centromeres (pericentomeres) are highly heterochromatic. In the present study, a total of 43 contigs were mapped within a genomic region represented by deletion bins of C-6BS1, C-6BL12 and 6BL12, where the centromere of wheat chromosome 6B should be located (Fig. 5). A number of centromere-specific sequences have been reported for wheat and its relatives, including 15 Ty3/*gypsy*-type centromeric retrotransposons in wheat (*CRW*) [50], one centromeric satellite-like repeat (contig310431) in wheat [51], one Ty3/*gypsy* retrotransposon (*CCS1-R11H-2*) in *Ae. tauschii* [52], and one DNA marker sequence (KF719092) in rye. Using the 18 centromere-specific sequences described above, we performed a BLAST search against the BAC-based assembled genomic sequences obtained from all of the above contigs (unpublished data). A total of 17 sequences (a-q shown in Fig. 5) were detected within the different MTP BAC clones from 17 contigs that were physically located within the above three deletion bins. In particular, eight of these contigs were found to carry at least three Ty3/*gypsy*-type sequences (*CRW* or *CCS1-R11H-2*), indicating a significant accumulation of Ty3/*gypsy* retrotransposons in these genomic regions, which is consistent with results reported for other wheat chromosomes [51, 53]. The results obtained from the mapping and sequencing of these contigs clearly indicate that they contain a portion of the centromeric region of wheat chromosome 6B (Fig. 5). Contig CTG125 (0.5 Mb), located proximally on the short arm of chromosome 6B, contains eight centromere-specific sequences, whereas six and four centromere-specific sequences were found within the two contigs CTG3378 (1.0 Mb) and CTG3387 (0.8 Mb), which mapped to the proximal region of the long arm (Fig. 5; Additional file 5). Many centromeric sequences were detected within other contigs (CTG14, CTG2884 and CTG9 on 6BS [bin C-6BS1, 40 cR from the boundary between 6BS and 6BL], and CTG287 on 6BL [bin 6BL12, 48.6 cR from the boundary]) (Fig. 5; Additional file 5). These results suggest that the genomic region from contig CTG14 to contig CTG287, with an estimated physical size of 25.4 Mb (10 contigs with 13.0 Mb on 6BS and 14 contigs with 12.4 Mb on 6BL), might correspond to the centromere of wheat chromosome 6B. However, we did not find any contigs with dominant satellite tandem repeats [51] in our physical map, although we detected sequences for the centromeric satellite-like repeat in CTG2884 on 6BS. A possible explanation for this observation is that the postulated ‘centromere core region’ with dominant satellite tandem repeats was not cloned or assembled because of its highly repetitive sequence composition.

A shift in the position of the centromere since the divergence of wheat and rice has been suggested based on comparisons between wheat chromosome 3B and rice chromosome 1 [51]. To dtermine whether the centromeric shift occurred between chromosome 6B and rice chromosome 2, we investigated the relationships of orthologous genes in the centromeric region between wheat 6B and rice 2. A total of 54 genic markers were mapped on the contigs at the centromeric deletion bins (C-6BS1, C-6BL12 and 6BL12), of which 47 markers were associated with pericentromeric rice genes on chromosome 2 (Fig. 5). This result supports a conservation of the centromeric region, as shown in a previous study [54]. The centromeric region, from contig CTG307 (C-6BS1) to contig CTG482 (C-6BL12), was interposed between the genic markers TNAC8059 (CTG307 on C-6BS1) and GZ6H037 (CTG2358 on C-6BL12) (Fig. 5; Additional file 5). These two markers aligned to rice genes Os02g0241600 and Os02g0244700, respectively, on the short arm and at a position 5.6 Mb distal to the centromere of rice chromosome 2 (Fig. 5). This suggests that the shift in the position of the centromere occurred between wheat 6B and rice 2, as observed between wheat 3B and rice 1.

### Distribution of genes along wheat chromosome 6B

**Fig. 6:**
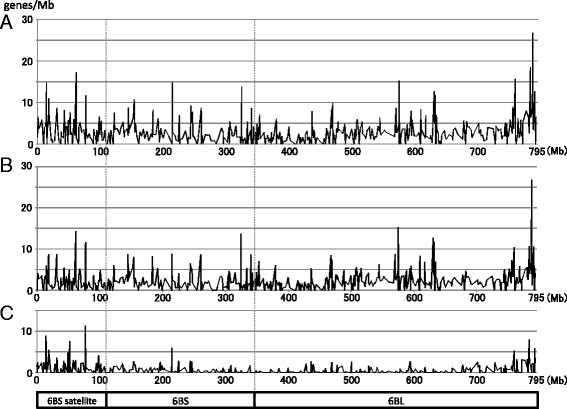
Gene density of each contig on chromosome 6B. The distribution patterns of gene density on each of the 480 contigs on chromosome 6B are indicated in genes/Mb. **a** The pattern of all 2015 genes assigned on the physical map. **b** The pattern of 1538 syntenic genes, compared to those of rice chromosome 2, *B. distachyon* chromosome 3 or sorghum chromosome 4. **c** The pattern of 477 nonsyntenic genes

Genes are not evenly distributed along wheat chromosomes [18, 19, 55, 56]. After the removal of redundant markers based on sequence similarity, we discovered 875 markers derived from independent genes (378 on 6BS and 497 on 6BL), which were able to anchor 322 contigs (137 on 6BS and 185 on 6BL). To further extend the substantial gene content on chromosome 6B, we applied the gene models from the survey sequence data [15]. The sequence contigs assembled from the 6B survey sequencing were mapped to the corresponding BAC contigs using their sequence similarities with the WGP tag sequences by BLAST. As a result, 50.9 and 61.1 % of 6BS tags and 6BL tags were mapped on 21,339 IWGSC 6BS sequence contigs (80.5 Mb) and 29,957 IWGSC 6BL sequence contigs (70.1 Mb). Finally, we mapped 1696 annotated genes on the survey sequence contigs to the physical map. After removal of redundancy, 1140 genes were integrated into the analysis based on the survey sequence data. In total, 2015 independent genes (859 on 6BS and 1156 on 6BL) were assigned to 395 contigs (170 on 6BS and 225 on 6BL). Based on the chromosomal locations of these genes on the contigs aligned along the RH map (Additional file 5), we obtained insight into the gene distribution pattern along chromosome 6B (Fig. 6a). The calculation of the numbers of genes and physical sizes of the gene containing contigs revealed an average gene density with similar values of 3.54 and 3.57 gene/Mb for the two chromosome arms of 6BS and 6BL, respectively. However, the average gene densities for each contig were different; they ranged from 0.25 to 26.72 genes/Mb that were observed on the pericentromeric contig CTG42 on 6BS and the telomeric contig CTG3358 on 6BL, respectively. The gene densities tended to increase from the centromeric to telomeric regions along chromosome 6B (Fig. 6a). This result was similar to the gene distribution on chromosomes 1B and 3B based on information obtained for deletion bins or the currently completed genomic sequences [18, 19, 55, 56].

On chromosomes 1B and 3B, the distribution of syntenic genes had no impact on the overall gene-density gradient [18, 19, 55]. We found that 1538 of 2015 genes assigned on chromosome 6B had orthologs on rice chromosome 2, *B. distachyon* chromosome 3 or sorghum chromosome 4 and the remaining 477 genes were nonsyntenic. The distribution patterns of the nonsyntenic group on 6BS showed a clear gradient of gene density, becoming increasingly dense from centromere to telomere, whereas there was no discernable gradient pattern for the syntenic group (Fig. 6b and c). This shows that the gradient of gene density along 6BS is due to the presence of nonsyntenic genes, which was consistent with the general pattern of gene density on wheat chromosomes 1B and 3B [18, 19, 55]. In contrast, on 6BL, both the nonsyntenic and syntenic genes increased in density from centromere to telomere (Fig. 6b and c). This result suggests that the general pattern discussed above may not apply to 6BL. However, our result did not completely cover the positions of the genes on the 6B physical map; we used 2015 genes, representing only half of the 3746 high-confidence genes annotated on chromosome 6B based on the IWGSC survey sequencing data [15]. Completion of the genomic sequencing and gene annotation will reveal the gene organization on chromosome 6B more definitively.

### Local rearrangements on wheat chromosome 6B

**Fig. 7:**
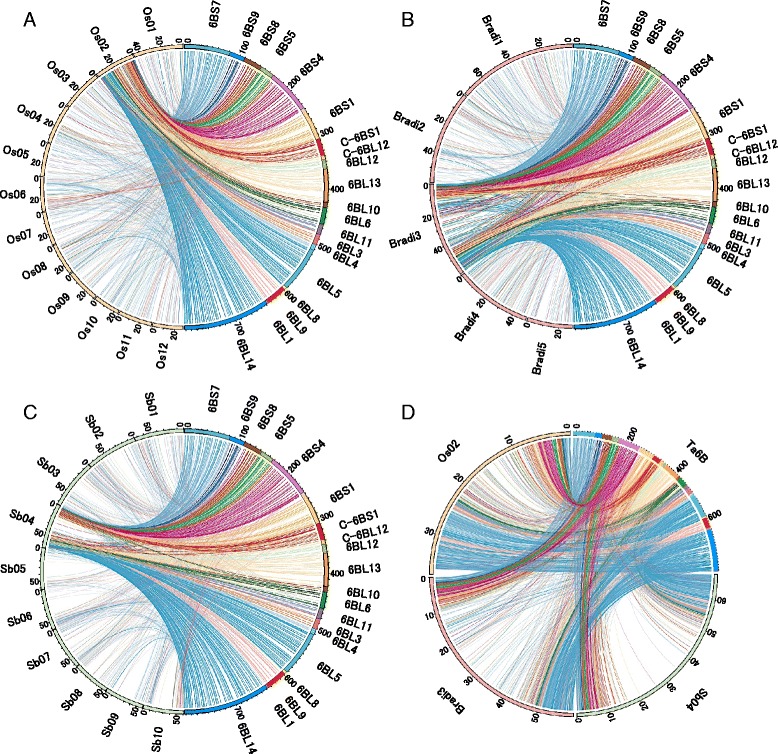
Shared synteny between wheat chromosome 6B and the chromosomes in rice, *B. distachyon* and sorghum. Wheat chromosome 6B is indicated on the right side of the circle, and each color corresponds to a deletion bin shown in Fig. 3. The chromosomes of **a** rice (Os01-12), **b** *B. distachyon* (Bradi1-5) and **c** sorghum (Sb01-10) are represented on the left side of the circle. **d** Comparisons between wheat chromosome 6B (Ta6B) and the homologous chromosomes (Os02, Bradi3 and Sb04). The lines represent the relationships between mapped genes on the 6B physical map and the orthologous genes in rice, *B. distachyon* and sorghum. The order of wheat genes in a given deletion bin is predicted by the alignment of contigs along the RH map

Previous comparative studies have demonstrated a syntenic and evolutionary relationship between the wheat homoeologous group 6 chromosomes and rice chromosome 2 (Os02), *B. distachyon* chromosome 3 (Bradi3) or sorghum chromosome 4 (Sb04) [42, 57–59]. Of the 2015 genes used in the present study for chromosome 6B, 1150, 1415 and 1172 genes were syntenic with those of Os02, Bradi3 and Sb04, respectively, revealing strong genome colinearity between wheat and other grass species (Figs. 7a–c, respectively). However, the present study identified several specific genome rearrangements caused by events, such as inversions or translocations, as well as a lack of colinearity resulting from insertions and deletions.

Two inversions (188.3–226.9 and 230.2–267.1 Mb on the physical map, as shown in Additional file 5) were detected between wheat 6BS and the three syntenic chromosomes, Os02, Bradi3 and Sb04 (Table 6; Additional file 7), providing strong evidence for evolutionary events specific to the wheat lineage. However, between chromosome 6B and rice Os2, two additional inversions were observed on the long arm (516.9–555.0 and 632.1–650.0 Mb), one of which (1205.7–1286.0 cR) was also identified between wheat and *B. distachyon* (Table 6; Additional file 7A and B). This finding suggests that the inversion involving chromosomal region 516.9–555.0 Mb occurred recently, after the divergence of Brachypodieae and Triticeae. Interestingly, the other inversion on the long arm (632.1–650.0 Mb) shared by wheat chromosome 6B and Bradi3 but not observed on Os02 was detected between chromosome 6D of *Ae. tauschii* and Os02 (Luo et al. [60]), suggesting that this event occurred after the divergence of Ehrhartoideae (Oryzae) and Pooideae (Brachypodieae and Triticeae) and before the divergence of Pooideae. A comparison of wheat chromosome 6B with Bradi3 indicated several complex rearrangements that appeared to have occurred within the genomic region of its long arm ranging from the deletion bin 6BL9 to the proximal two-fifth part of 6BL14 (Table 6; Additional file 5). This region included several translocations and inversions (602.5–713.8 Mb on the physical map), as well as a reciprocal translocation between the two regions on 602.5–713.8 and 715.8–786.4 Mb. These translocations might be Brachypodieae-specific (Table 6; Additional file 7B) because the synteny between 6B and Os02 in this region was well conserved (Additional file 7A).

**Table 6:** Genomic regions containing chromosome rearrangements between 6B and its homologous chromosomes in other species

Wheat chromosome 6B	Wheat chromosome 6B	Rearrangement	Rice chromosome 2	*B. distachyon* chromosome 3	Sorghum chromosome 4
(position on RH map)	(estimated size ^a^)		(size)	(size)	(size)
6BS: 188.3–226.9 Mb	6BS: 355.7–422.8cR	Inversion	Os02g0169900-Os02g0184500	Bradi3g05260.1-Bradi3g06250.1	Sb04g004700.1-Sb04g005730.1
(355.7–422.8cR)	(41.9 Mb)		(0.93 Mb)	(0.85 Mb)	(1.1 Mb)
6BS: 230.2–267.1 Mb	6BS: 424.0–511.9cR	Inversion	Os02g0185500-Os02g0200000	Bradi3g06260.1-Bradi3g07370.1	Sb04g005790.1-Sb04g006830.1
(424.0–511.9cR)	(40.4 Mb)		(0.84 Mb)	(0.94 Mb)	(1.2 M)
6BL: 516.9–555.0 Mb	6BL: 1205.7–1286.0cR	Inversion	Os02g0644100-Os02g0680400	Bradi3g50100.1-Bradi3g51730.1	
(1205.7–1286.0cR)	(43.8 Mb)		(1.9 Mb)	(1.4 Mb)	
6BL: 632.1–650.0 Mb	6BL: 1400.4–1432.3cR	Inversion	Os02g0739000-Os02g0751800		
(1400.4–1432.3cR)	(22.3 Mb)		(0.73 Mb)		
6BL: 602.5–713.8 Mb	6BL: 1373.4–1479.1cR	Translocation (inversion)		Bradi3g56180.1-Bradi3g60210.1	
(1373.4–1479.1cR)	(98.8 Mb)			(3.0 Mb)	
6BL: 715.8–786.4 Mb	6BL: 1494.5–1545.2cR	Translocation		Bradi3g52910.1-Bradi3g56110.1	
				(2.4 Mb)	
(1494.5–1545.2cR)	(70.6 Mb)				
6BL: 516.9–555.0 Mb	6BL: 1205.7–1286.0cR	Translocation			Sb04g031850.1-Sb04g033430.1
(1205.7–1286.0cR)	(43.8 Mb)				(1.6 Mb)
6BL: 559.5–630.8 Mb	6BL: 1293.0–1398.2cR	Inversion			Sb04g028600.1-Sb04g031680.1
(1293.0–1398.2cR)	(72.4 Mb)				(2.9 Mb)
6BL: 632.1–650.0 Mb	6BL: 1400.4–1432.3cR	Translocation			Sb04g027710.1-Sb04g028500.1
(1400.4–1432.3cR)	(22.3 Mb)				(0.96 Mb)

^a^The size of the genomic region on chromosome 6B is estimated based on the cumulative size of the BAC contigs positioned in the corresponding RH map region

A comparison of wheat chromosome 6B with Sb04 led to the discovery of one large inversion (559.5-630.8 Mb on the physical map) of the long arm 6BL (Table 6; Additional file 7C). Because wheat 6B and Os02 or Bradi3 share the same inversion relative to Sb04, this chromosomal structural change might be unique to the sorghum lineage. We discovered two reciprocal translocations between wheat 6B and Sb04 (Table 6; Additional file 7C), which corresponded exactly to the two Pooideae-specific inversions described above (Table 6; Additional file 7A).

**Fig. 8:**
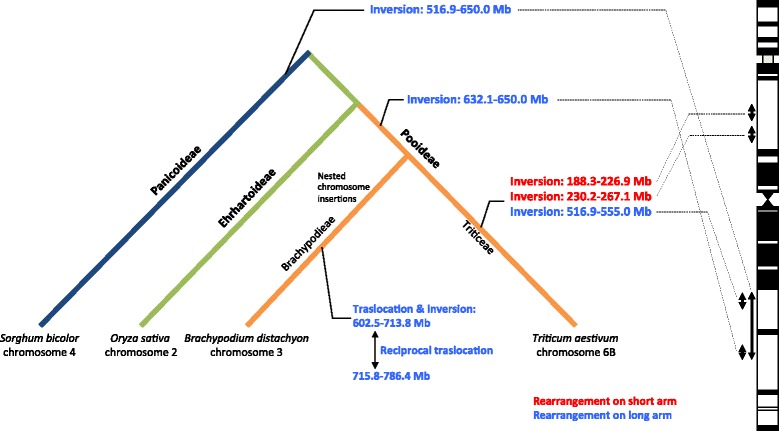
Evolutionary events underlying the chromosomal rearrangements on 6B and the homologous chromosomes in other species. A phylogenetic tree representing the grass subfamilies Panicoideae, Ehrhartoideae and Pooideae is shown. Pooideae includes the additional subgroups Brachypodieae and Triticeae. The estimated time point for each rearrangement event is indicated by a black line on the tree branch. The chromosomal region of each rearrangement is indicated by its physical length (Mb) based on the 6B physical map. Rearrangements on the short and long arms are represented by red and blue characters, respectively. The chromosomal regions of each inversion are represented by arrows on the left side of the C-banding karyotype of chromosome 6B

These data allow us to propose the following scenarios for rearrangements on the long arm during the evolution of grass species (Fig. 8): 1) the chromosomal region corresponding to 516.9–650.0 Mb on the physical map was inverted after the divergence of Panicoideae (*Sorghum*) and Ehrhartoideae/Pooideae; 2) the distal part of the first inversion, 632.1–650.0 Mb, was inverted again after the divergence of Ehrhartoideae and Pooideae; and 3) the proximal region of the first inversion, 516.9–555.0 Mb, was recently inverted after the divergence of Brachypodieae and Triticeae. These results also indicate that chromosomal rearrangements occurred frequently in the distal regions of the homologous chromosomes, including wheat 6B.

### Disruption of synteny in the proximal region of wheat chromosome 6BL

A large rearrangement in the proximal region (deletion bins, C-6BL12, 6BL12 and 6BL13; Fig. 7d; 340.2–421.8 Mb in Additional file 7; Additional file 5) was observed between 6B and the Sb04, Os02, and Bradi3 chromosomes; that is, fewer syntenic genes were mapped to this genomic region. Such an interruption of synteny in the proximal region of wheat homoeologous group 6 chromosomes relative to rice chromosome Os02 has been previously described by the mapping of orthologous genes [42]. It has also been documented between chromosome 6D of *Ae. tauschii* and Os02 based on high-resolution physical mapping [60]. Using the genomic sequences of the MTP BAC clones in this region (unpublished results), we estimated the number of genes in these bins that were homologous to the rice genes by a BLAST analysis. From 864 MTP clones (85 Mb) in the three bins, we identified 517 gene sequences homologous to the rice genes. We found 70 and 69 gene sequences that were homologous to Os01 and Os02, respectively, and 25–56 gene sequences were derived from other chromosomes in rice. We investigated the colinearity of the 69 sequences that were homologous to Os02 based on their positions on Os02, but we failed to detect a certain level of colinearity. The estimated proportion of nonsyntenic genes in this region was very high (87 %; 449 in 517 sequences) compared with the proportion (57 %) in the entire chromosome 6B based on our survey sequence data, as noted previously [24]. Furthermore, a recent genome sequencing study reported that the proportion of nonsyntenic genes in the proximal region of chromosome 3B was 28 %, and even in distal regions, the proportions were 44 and 53 % [56]. These results suggest that the interruption was unusual; it was not due to the simple integration of chromosomal segments from a rice chromosome other than Os02 into this region but rather to the abundant and random accumulation of nonsyntenic genes that might disrupt the synteny between wheat chromosome 6B and rice chromosome 2. Although we do not currently have any data to explain the mechanisms underlying the disruption in colinearity in this region, it might be due to structural differences in the centromeric region, including the previously described centromere shifting.

Large interruptions of approximately 30–40 Mb of the genomic region of Bradi3 and Sb04 were also found (Fig. 7d; Additional files 7B and C). The interruption between 6B and Bradi3 revealed characteristics of the chromosomal structures of Bradi3, which was postulated to correspond to three rice chromosomes (Os02, Os08 and Os10), and its pericentromeric 10–45 Mb region was syntenic with Os08 and Os10 [6]. Therefore, the interruption between 6B and Bradi3 resulted from the insertion of other chromosomal segments in Bradi3. The cause of the interruption between 6B and Sb4 remains unclear, although the small number of annotated gene in this 30-Mb genomic region corresponding to the proximal region of Sb04 [7] is estimated to be one of the causes.

In summary, we could infer the structural features of chromosome 6B with high resolution based on the the physical localizations of genic markers obtained by RH mapping (Fig. 3e). The results provide information to understand the molecular and biological mechanisms underlying the genomic divergence and chromosomal evolution of wheat.

## Conclusions

Here, we provide a BAC-based physical map of wheat chromosome 6B with an estimated size of 833.8 Mb that covers 91 % of the chromosome, which allowed us to select MTP BAC clones for the map-based genomic sequencing of chromosome 6B. The development of 2860 anchor markers and the construction of a high-resolution RH map permitted the successful localization of 480 of the assembled contigs to their chromosomal regions, representing 87 % (794.8 Mb) of the entire chromosome. The establishment and analysis of the integrated physical map also led to the discovery of several important features of chromosome 6B, including the fine chromosomal localization and organization of *Nor-B2* and the centromere, the gene distribution patterns and the evolutionary history of chromosomal rearrangements among grass species. Furthermore, the use of marker information from syntenic genes led to the identification of chromosomal regions that were highly conserved or frequently rearranged, which is useful for our understanding of the complexity of genome evolution in wheat.

## Methods

### Construction of BAC libraries

The short and long arms of wheat chromosome 6B were isolated from a double ditelosomic line (2n = 40 + 2t6BS + 2t6BL) of CS [30] that was obtained from NBRP-Wheat Japan (accession number LPGKU0038). Aqueous suspensions of intact mitotic chromosomes were prepared from synchronized root tips of young seedlings as described by Vrána et al. [61], and both arms of 6B were purified by flow cytometry [24]. The purity of the flow-sorted fractions was determined by FISH using probes for GAA microsatellites and telomeric repeats as described by Janda et al. [62]. A total of 5,200,000 6BS arms and 5,150,000 6BL arms were sorted by flow cytometry, embedded in agarose miniplugs (each containing approximately 200,000 arms) and used to construct BAC libraries according to Šimková et al. [63]. Briefly, high-molecular-weight DNA was partially digested with *Hin*dIII and subjected to two rounds of size selection using pulsed-field gel electrophoresis. The DNA was electroeluted from the gel and ligated into the pIndigoBAC-5 vector (Epicentre, Madison, WI, USA). The recombinant vector was used to transform *Escherichia coli* DH10B competent cells (Invitrogen, Carlsbad, CA, USA). The libraries were ordered into 384-well plates filled with freezing medium consisting of 2YT, 6.6 % glycerol and 12.5 μg/ml chloramphenicol and stored at −80 °C. The average insert size was estimated based on an analysis of 240 randomly selected BAC clones in each library.

### Fingerprinting by whole-genome profiling

Fingerprinting of chromosome arm 6BS- and 6BL-specific BAC libraries was performed by using a WGP™ method that was developed based on NGS technology to establish the chromosome physical maps [31]. Using 41,472 clones of 6BS (plate no. 41–148) and 49,920 clones of 6BL (plate no. 69–198), a multi-dimensional pool of BAC DNAs with a high concentration and low *E. coli* level was developed by Amplicon Express, Inc. (Pullman, WA, USA). The pooled BAC DNAs were subjected to sample preparation and sequencing by KeyGene N.V. (Wageningen, The Netherlands): a) digestion with the restriction enzymes *Eco*RI and *Mse*I, ligation of sequencing adaptors containing sample identification tags and PCR amplification; b) pooling of the PCR products; c) cluster amplification; and d) sequencing from the *Eco*RI side using the HiSeq2000 sequencer (Illumina, San Diego, CA, USA) with a read length of 81 nucleotides. The high-quality reads were used for WGP data processing, which included the following steps: a) deconvolution, i.e., assignment of sequencing reads to individual BACs in the pools; b) assignment of WGP tags to BACs based on the deconvoluted reads; and c) filtering of WGP tags and BACs using various quality control measures to minimize noise.

### BAC contig assembly

The assembly of BAC clones was performed using FPC software [33], which was improved by KeyGene N.V. to be capable of processing the WGP data. The assembly procedure was based on the previously described guidelines for physical map assembly of IWGSC and chromosome 3B physical mapping [20, 32] as shown below: a) the initial assembly was performed using incremental contig-building with a cut-off of 1e^−75^; b) a single-to-end and end-to-end merge was conducted using the Match 1 parameter by increasing the cut-off (1e^−5^ at each step) to a final cut-off of 1e^−05^; c) the DQer function was used at each cut-off step to separate all of the contigs containing more than 10 % of Questionable (Q) clones with the Step 3 parameter; d) finally, a single optimal cut-off value was determined. The following parameters were used for the WGP analysis: a band size (CB unit, average distance between tags) of 5220 and 5075 for 6BS and 6BL, respectively; a gel length (corresponding to the number of unique tags) of 120,913 and 112,559 for 6BS and 6BL, respectively; a FromEnd value (corresponding to half of the average number of tags per BAC) of 12; and a tolerance of 0. The MTP was selected using the FPC MTP module [64].

### Elimination of low-confidence BAC contigs

To detect BAC clones derived from other chromosomal DNAs that contaminated our preparation, a BLASTN [65] search was performed against the survey sequence of wheat [15] using the WGP tag sequences on the BAC clones as queries with thresholds of 95 % coverage and 95 % identity. First, we identified WGP tags with high similarity to each chromosome group. Next, we compared the number obtained for the group 6 chromosomes and the largest number among the other chromosome groups. If the number obtained for one of the other groups was larger (more than four) than the number obtained for group 6, we judged that the BAC clone was derived from the other chromosomes. Contigs that included BAC clones occupying more than 66 % of the total number of BAC clones within a contig were eliminated from further experiments and were labeled as low-confidence contigs.

### Marker selection and development

We used several sources to develop markers used in this study, which were mainly divided into three groups. The first group was the markers that genetically mapped to chromosome 6B and deposited in the public databases: GrainGenes CMap and NBRP-Wheat, Japan. From these databases, we selected 208 PCR-based markers such as STS-RFLP and SSR markers. Additionally, 751 PLUG markers [34, 35] (http://plug.dna.affrc.go.jp/) were selected based on synteny of wheat group-6 chromosomes to rice chromosome 2.

The second group consisted of markers derived from wheat and barley expressed sequences, i.e., full-length cDNAs [66, 67] and ESTs (Ta#56: wheat ESTs deposited in build #56 of the NCBI UniGene dataset; http://www.ncbi.nlm.nih.gov/unigene). In the Genome Zipper [36], we found 2304 non-redundant reads by NGS mapped on chromosome 6H of barley. We identified wheat ESTs homologous to the above barley 6H reads by BLAST (390 markers). Additionally, we selected following wheat sequences as marker sources: a) full-length cDNAs with ≥70 % coverage and ≥50 % identity with the syntenic genes common to Os02, Bradi3 and Sb04 (orthologous set, 905 markers); b) full-length cDNAs and ESTs with more than 80 and 85 % identities to genes of Os02 and Bradi3, respectively (predicted orthologous set, 482 FLcDNA and 309 EST markers); and c) full-length cDNAs with more than 85 % identity with Bradi3 genes listed in the barley 6H Genome Zipper (predicted orthologous set from *B. distachyon*, 133 markers).

The third group contained ISBP markers, which were developed from the survey sequence data for 6BS and 6BL [15] according to Paux et al. [28]. Among the high-confidence primer set identified (Kaneko et al. in preparation), 2000 sets were selected based on the frequency of the number of junctions between the transposable element subfamilies.

To determine the chromosomal locations of contigs that lacked the above anchors, additional PCR markers such as ISBP and SSR were developed using the genomic sequences of the MTP BAC clones and were used for RH mapping. High-confidence ISBP markers were developed as described by Paux et al. [28], and Primer3 was used to design SSR primers at positions flanking the microsatellite sequences (>4 units of di-, tri- and tetra-nucleotides) predicted by Perl scripts. To develop 6B-specific markers, a BLASTN search using the primer sequences as queries was performed against the wheat survey sequence data [15]. Primer pairs with complete identity to the only chromosome 6B sequences were 1076 ISBP (426 for 6BS and 650 for 6BL contigs) and 1163 SSR (447 for 6BS and 716 for 6BL contigs) markers, from which 79 ISBP (37 for 6BS and 42 for 6BL contigs) and 74 SSR (38 for 6BS and 36 for 6BL contigs) were used for anchoring.

### PCR screening of BAC libraries

Prior to BAC library screening, the PCR-based molecular markers were tested to determine their quality and specificity to chromosome 6B. For the PCR amplification, total DNA was extracted from CS, the Nullisomic-6B Tetrasomic-6A (N6BT6A) line and M808 using standard procedures, and the DNAs of the sorted chromosome arms 6BS and 6BL were amplified by multiple displacement amplification using the illustra™ GenomiPhi V2 DNA Amplification Kit (GE Healthcare Bio-Science Corp., Piscataway, NJ, USA). PCR reactions were performed using Quick Taq Solution (Toyobo, Osaka, Japan) according to the manufacturer’s recommendations. Touchdown PCR amplification was performed as follows: initiation at 94 °C for 2 min, 10 cycles (94 °C for 20 s, 65 °C minus 0.5 °C each cycle for 20 s, 72 °C for 20 s), 20 cycles (94 °C for 2 min, 60 °C for 20 s, 72 °C for 20 s) and termination at 72 °C for 1 min. The amplified products were analyzed by using a MultiNA microchip electrophoresis system (Shimadzu, Kyoto Japan) according to the manufacturer’s recommendations.

Screening was performed using the BAC DNA pools, which were created from the above BAC libraries for 6BS and 6BL, comprising 57,600 and 76,032 BAC clones, respectively. The BAC DNAs were extracted from three-dimensional (plate, row, column) pooled BACs and purified using the alkaline lysis method [68]. Each pool contained 100 μg of BAC DNA. The PCR reaction was performed in 15 μL containing 30 ng of BAC pooled DNA, 0.2 μM of each primer and 5 μL of GoTaq Green Master Mix (Promega, Madison, WI, USA). Amplification was initiated at 94 °C for 1 min, followed by 35 cycles of 94 °C for 30 s, 60 °C for 1 min, 72 °C for 1 min, and termination at 72 °C for 1 min. The resulting fragments were electrophoresed in a 2 % agarose gel.

### RH mapping

The RH panel of chromosome 6B was produced by Watanabe et al. (in preparation). We selected 355 lines as the RH panel based on the genotypes determined using the 21 6B-specific SSR markers. In addition to the RH panel, 21 chromosome deletion lines (9 lines for 6BS and 12 lines for 6BL) [39] were used for the approximate mapping of markers to each chromosome bin. CS and the N6BT6A line were included as positive and negative controls, respectively, for the amplification of the PCR markers. The seeds of CS (LPGKU2269), the N6BT6A line (LPGKU0075) and the 21 chromosome deletion lines (LPGKU1247-1267) were obtained from NBRP-Wheat, Japan.

To genotype the RH panel and chromosome deletion lines, we used 653 markers that were anchored on 462 contigs and defined based on their specificity for chromosome 6B using the N6BT6A line. The presence/absence of the markers was analyzed by agarose gel electrophoresis.

The data obtained for the 653 markers were used to group and order the markers in Carthagene version 1.3.beta [69]. The linkage group was determined by using the group command with a two-point LOD threshold of 4.0 and a maximum distance of 100 cR. The heap command was used to identify the map with the highest likelihood. The mapocb command was used to calculate the number of obligate breaks.

### Calculation of gene density along chromosome 6B

We used two gene information sources to calculate the gene density along chromosome 6B. First, we extracted markers representing independent genes from the DNA markers used for the anchoring BAC clones. Redundancy in the marker set was removed based on the sequence similarity using CAP3 [70] with 98 % identity. BLASTX search for the resulting non-redundant markers were conducted to the rice, *Brachypodium* and sorghum genes with E value < 10^−5^. Then, markers hit to the genes on rOs02, Bardi3 and Sb04 were used for the further analysis. Second, we used gene models annotated from the survey sequencing data by IWGSC [15]. We mapped 731,925 and 998,825 WGP tags on 6BS and 6BL to IWGSC sequence contigs assembled from the 6B survey sequencing [15] by megablast with default setting. Gene information on 6B survey sequence contigs were assigned to BAC contigs based on the information of the WGP tags uniquely mapped to 6B survey sequence contigs with 100 % identity and 100 % coverage. If the tags located in distant contigs were mapped on a same survey sequence contig, we discarded the hit information.

### Comparison of the virtual gene order based on the physical map with the syntenic chromosomes of other cereals

To compare the gene order between 6B and its homologous chromosomes, Os02, Bradi3 and Sb04, a BLASTX search with an E-value < 10^−5^ [65] was conducted against the protein sequences of the three grasses using nucleotide sequences of the aforementioned gene set. The positions of the genes on the 6B physical map were compared with the physical genomic positions of the orthologous sequences on the homologous chromosomes in the three cereals. The graphical displays of the relationships between orthologous genes were drawn using Circos-0.67-7 software [71].

### Sequencing of MTP BAC clones

BAC clones were sequenced using Roche GS FLX (Basel, Switzerland). BAC DNAs were prepared in a 96-deepwell plate, extracted using the alkaline lysis method and purified with the MultiScreen Filter plate (Millipore, Billerica, MA, USA). After fragmentation with the Covaris Acoustic Solubilizer LE220 (Covaris, Woburn, MA, USA), the sequencing libraries were constructed using the NEBNext DNA Library Prep Master Mix Set for 454 (New England Biolabs, Ipswich, MA, USA) and tagged with Multiplex Identifier (MID) Adaptors (Roche, Basel, Switzerland). The tagged DNAs were subjected to size selection using agarose gel electrophoresis to obtain DNA fragments of the appropriate size (600–1200 bp) and then quantified using the Agilent 2100 Bioanalyzer (Agilent, Santa Clara, CA, USA). The resulting libraries were sequenced according to the manufacturer’s recommendations. The resulting SFF files were split into separate files of each BAC clone based on MID by using the sffile program in SFF Tools (Roche, Basel, Switzerland). The split SFF files were used for *de novo* assembly with the GS *De Novo* Assembler v2.6 (Roche, Basel, Switzerland) with default parameters, and the BAC vector and *E. coli* genome sequences were trimmed.

### Sequence analysis

To detect ribosomal RNA genes, a BLASTN search was performed against BAC sequences with E-values < 10^−10^. For use as queries, four rRNA gene sequences from wheat, 5S (accession no. 3IZ9), 5.8S (3IZ9), 18S (3IZ7) and 25S (3IZ9) [72], were obtained from the Protein Data Bank (PDB; http://www.pdb.org/pdb/home/home.do), and the IGS sequence (X07841) [44] was obtained from DDBJ/EMBL/GenBank (http://www.ddbj.nig.ac.jp).

To identify BAC clones containing centromeric sequences, a BLASTN search was run against the BAC sequences with E-value < 10^−4^, and a low-complexity filter was used. Eighteen centromeric sequences were used as queries: 15 probe sequences (KC290868-KC290916) [50], the *CCS1-R11H-2* gene (AB048245) [52], the rye centromere-specific marker (KF719092) and contig 310431 [51] of the 5xCS genome sequence [11].

## Availability of supporting data

A genome browser of the physical map of the wheat chromosome 6B is available from the Unité de Recherche Génomique Info website (https://urgi.versailles.inra.fr/gb2/gbrowse/wheat_phys_pub/) and the Komugi Genome Sequence Program web site (http://komugigsp.dna.affrc.go.jp/index.html). The other supporting data are included as additional files.

## Additional files

Additional file 1:
**BAC fingerprinting by whole-genome profiling.** (PDF 35 kb) Additional file 2:
**Distribution of the number of WGP tags per BAC clone in the libraries of 6BS and 6BL.** To fingerprint the BAC libraries with WGP, the number of tags on each clone was determined by deconvolution by assigning the sequence reads to individual BACs. The gray bars represent the number of eliminated BACs that were judged to be low quality based on the number of tags (less than 7 tags or more than 51 tags). The BAC clones represented by white bars were used for subsequent contig assembly. (PDF 50 kb) Additional file 3:
**Final fingerprinting data used for contig assembly after BAC filtering.** (PDF 33 kb) Additional file 4:
**Features of the physical maps of chromosome arms 6BS and 6BL.** (PDF 38 kb) Additional file 5:
**Integrated data for the RH map and BAC contigs of chromosome 6B.** The markers, distance between markers and cumulative distance are provided for the 6B RH map. The marker location on the deletion bin is also indicated. The ordered contig data mapped on the RH map with each of the markers provide the contig name, contig size, number of BAC clones, number of MTP clones and names of the markers anchored in the same contig. The physical position of each contig is denoted by the cumulative contig size starting from the contig ‘6BS_CTG219’ located on the end of 6BS. (XLSX 189 kb) Additional file 6:
**Schematic of the NOR structure by comparing wheat** ***Nor-B2*** **on chromosome 6B and rice NOR on chromosome 9.** The structure of the rice NOR on chromosome 9 is depicted according to Fujisawa et al. [49] with modifications. Wheat *Nor-B2* is illustrated based on Fig. 4, showing the five contigs located around the *Nor-B2* locus. Each rDNA unit is represented by a horizontal arrow in the illustration of rice and wheat. In the illustration of *Nor-B2*, ISBP and genic markers that mapped to the five contigs are indicated by white and black boxes, respectively. The junction sequences of the ISBP markers are indicated in parentheses below the marker name. The uncharacterized genomic region within the contigs is represented by a dashed horizontal line. (PDF 68 kb) Additional file 7:
**Relationship of the gene positions between wheat chromosome 6B and the syntenic chromosomes of grass species.** Dot plot of the best matches between genes on wheat chromosome 6B and the syntenic chromosomes: (A) 6B vs. rice chromosome 2 (Os02); (B) 6B vs. *B. distachyon* chromosome 3 (Bradi3); and (C) 6B vs. sorghum chromosome 4 (Sb04). The horizontal axis in the dot plots indicates the position of assigned genes on the 6B physical map, and the vertical axis indicates the physical positions of genes on each syntenic chromosome. (PDF 259 kb)

## Abbreviations

BAC: Bacterial artificial chromosome
BLAST: Basic Local Alignment Search Tool
cR: Centiray
EST: Expression sequence tag
FISH: Fluorescence in situ Hybridization
FPC: FingerPrinted Contigs
Gb: Gigabase
IGS: Intergenic spacer sequence
ISBP: Insertion site-based polymorphism
IWGSC: International Genome Sequencing Consortium
kb: Kilobase
Mb: Megabase
MTP: Minimal tiling path
NGS: Next-generation sequencing
NOR: Nucleolus organizer region
PCR: Polymerase chain reaction
PLUG: PCR-based Landmark Unique Gene
RFLP: Restriction fragment length polymorphism
RH: Radiation hybrid
RIL: Recombinant inbred line
rDNA: Ribosomal DNA
rRNA: Ribosomal RNA
STS: Sequence-tagged site
SSR: Simple sequence repeat
WGP: Whole Genome Profiling
