# Supplementary material for: A high-resolution physical map integrating an anchored chromosome with the BAC physical maps of wheat chromosome 6B
Source: BMC Genomics. 2015 Aug 12;16(1):595. doi: 10.1186/s12864-015-1803-y (PMC4534020; doi:10.1186/s12864-015-1803-y)
Supplement: Additional file 1: — BAC fingerprinting by whole-genome profiling. (PDF 35 kb) [file 12864_2015_1803_MOESM1_ESM.pdf]

Additional file 1 BAC fingerprinting by whole-genome profiling

	6BS	6BL
Number of BACs tested	41,472	49,920
Coverage	13.2x	13.2x
High-quality reads	316.1 Mb	467.1 Mb
Deconvolutable reads	172.7 Mb	272.0 Mb
Number of tagged BACs	35,515	45,895
Coverage	11.3x	12.0x
Number of unique WGP tags	122,164	113,522
Average number of WGP tags/BAC	20.6	21.8
Average distance between WGP tags (CB unit)	6,000 bp	5,700 bp
