# Supplementary material for: A high-resolution physical map integrating an anchored chromosome with the BAC physical maps of wheat chromosome 6B
Source: BMC Genomics. 2015 Aug 12;16(1):595. doi: 10.1186/s12864-015-1803-y (PMC4534020; doi:10.1186/s12864-015-1803-y)
Supplement: Additional file 2: — Distribution of the number of WGP tags per BAC clone in the libraries of 6BS and 6BL. To fingerprint the BAC libraries with WGP, the number of tags on each clone was determined by deconvolution by assigning the sequence reads to individual BACs. The gray bars represent the number of eliminated BACs that were judged to be low quality based on the number of tags (less than 7 tags or more than 51 tags). The BAC clones represented by white bars were used for subsequent contig assembly. (PDF 50 kb) [file 12864_2015_1803_MOESM2_ESM.pdf]

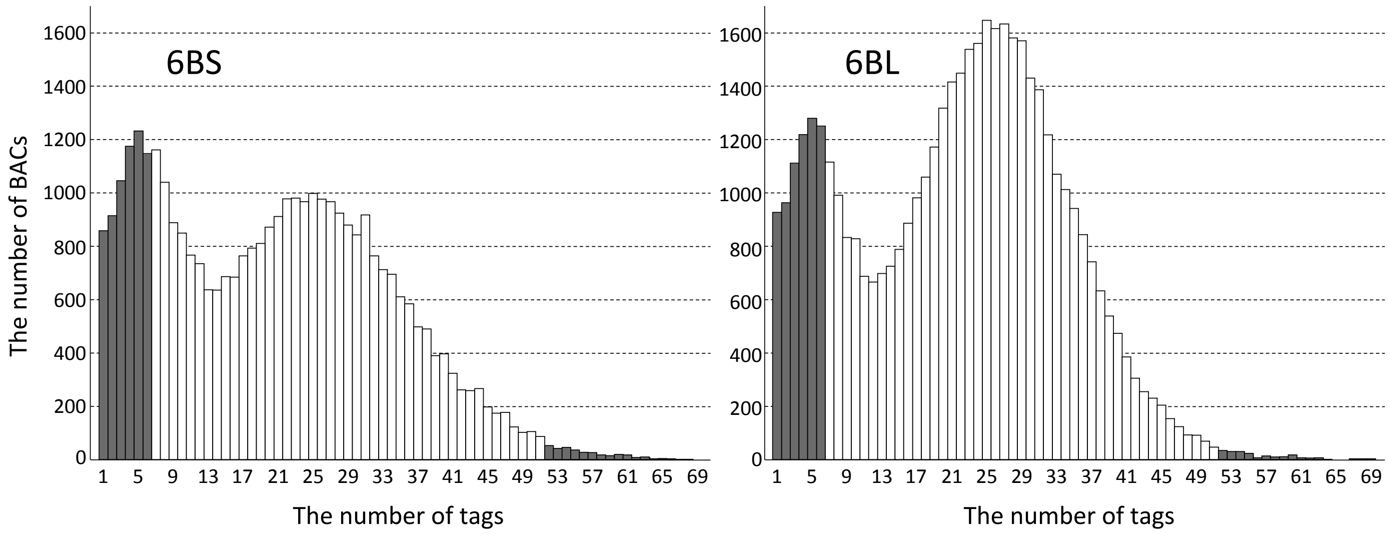

Additional file 2 Distribution of the number of WGP tags per BAC clone in the libraries of 6BS and 6BL.
To fingerprint the BAC libraries with WGP, the number of tags on each clone was determined by deconvolution by assigning the sequence reads to individual BACs. The gray bars represent the number of eliminated BACs that were judged to be low quality based on the number of tags (less than 7 tags or more than 51 tags). The BAC clones represented by white bars were used for subsequent contig assembly.
