# Supplementary material for: A high-resolution physical map integrating an anchored chromosome with the BAC physical maps of wheat chromosome 6B
Source: BMC Genomics. 2015 Aug 12;16(1):595. doi: 10.1186/s12864-015-1803-y (PMC4534020; doi:10.1186/s12864-015-1803-y)
Supplement: Additional file 3: — Final fingerprinting data used for contig assembly after BAC filtering. (PDF 33 kb) [file 12864_2015_1803_MOESM3_ESM.pdf]

Additional file 3 Final fingerprinting data used for contig assembly after BAC filtering

	6BS	6BL
Number of useful fingerprints	28,828	38,953
Coverage	9.2x	10.2x
Number of unique WGP tags	120,913	112,559
Average number of WGP tags/BAC	23.9	24.7
Average distance between WGP tags (CB unit)	5,220 bp	5,057 bp
