# Supplementary material for: A high-resolution physical map integrating an anchored chromosome with the BAC physical maps of wheat chromosome 6B
Source: BMC Genomics. 2015 Aug 12;16(1):595. doi: 10.1186/s12864-015-1803-y (PMC4534020; doi:10.1186/s12864-015-1803-y)
Supplement: Additional file 4: — Features of the physical maps of chromosome arms 6BS and 6BL. (PDF 38 kb) [file 12864_2015_1803_MOESM4_ESM.pdf]

Additional file 4 Features of the physical maps of chromosome arms 6BS and 6BL

	6BS	6BL
Assembly stringency	e-12	e-11
Number of BACs in the contigs	22,187	34,123
Number of singletons	6,640	4,829
Number of contigs	1,069	514
Estimated size (chromosome coverage*)	492 Mb (119%)	495 Mb (99%)
Average contig size (BACs)	21	66
Average contig size (kb)	461	963
Largest contig size (BACs)	327	587
Largest contig size (kb)	5,773	8,723
Contigs N50 (kb)	1,503	2,422
Contig L50	87	65

*Coverage was calculated based on the estimated size for each arm: 415 Mb for 6BS and 498 Mb for 6BL.
