# Supplementary material for: A high-resolution physical map integrating an anchored chromosome with the BAC physical maps of wheat chromosome 6B
Source: BMC Genomics. 2015 Aug 12;16(1):595. doi: 10.1186/s12864-015-1803-y (PMC4534020; doi:10.1186/s12864-015-1803-y)
Supplement: Additional file 6: — Schematic of the NOR structure by comparing wheat Nor-B2 on chromosome 6B and rice NOR on chromosome 9. The structure of the rice NOR on chromosome 9 is depicted according to Fujisawa et al. [49] with modifications. Wheat Nor-B2 is illustrated based on Fig. 4, showing the five contigs located around the Nor-B2 locus. Each rDNA unit is represented by a horizontal arrow in the illustration of rice and wheat. In the illustration of Nor-B2, ISBP and genic markers that mapped to the five contigs are indicated by white and black boxes, respectively. The junction sequences of the ISBP markers are indicated in parentheses below the marker name. The uncharacterized genomic region within the contigs is represented by a dashed horizontal line. (PDF 68 kb) [file 12864_2015_1803_MOESM6_ESM.pdf]

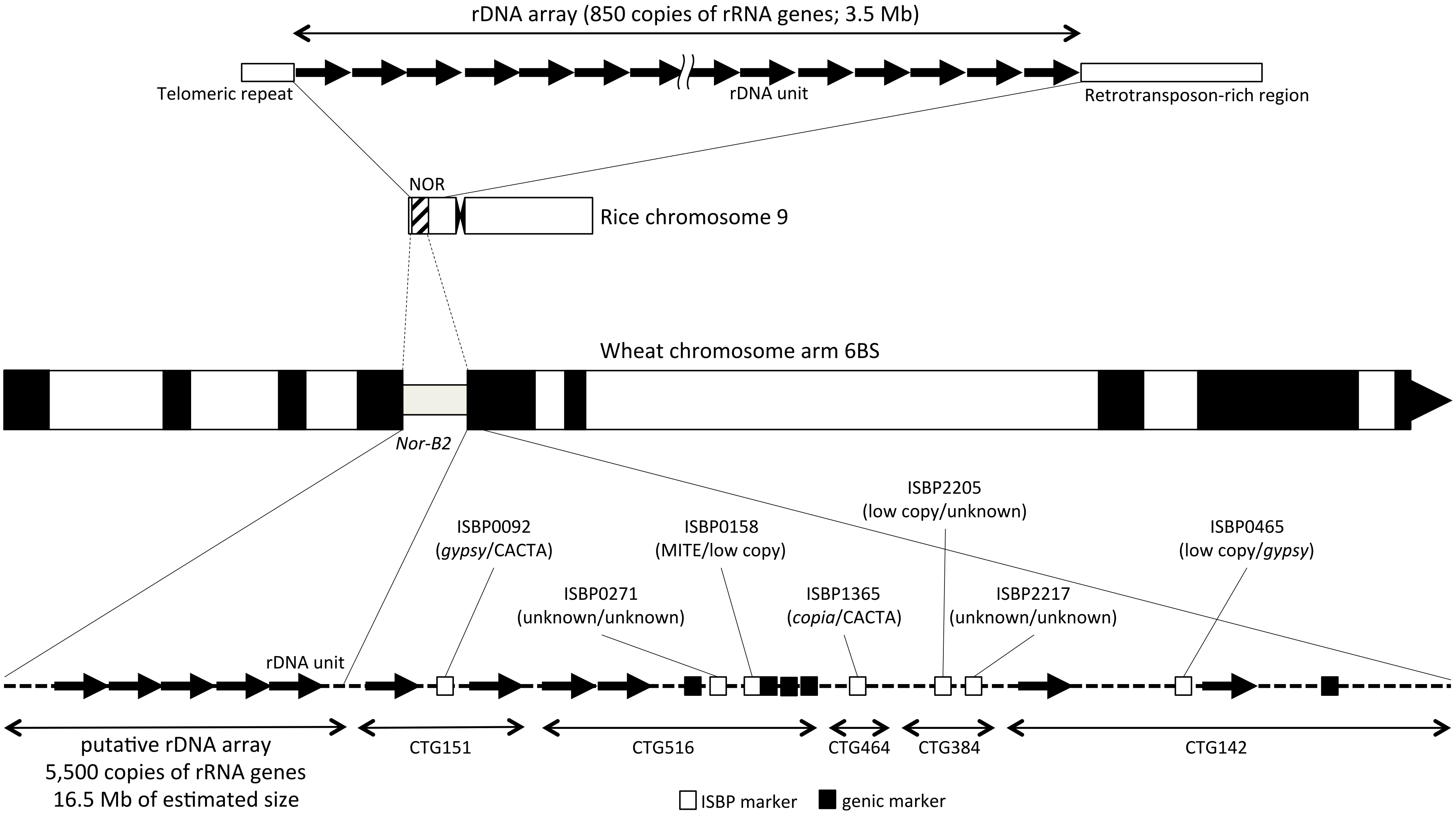

Additional file 6 Schematic of the NOR structure by comparing wheat *Nor-B2* on chromosome 6B and rice NOR on chromosome 9.
The structure of the rice NOR on chromosome 9 is depicted according to Fujisawa et al. [47] with modifications. Wheat *Nor-B2* is illustrated based on Figure 4, showing the five contigs located around the *Nor-B2* locus. Each rDNA unit is represented by a horizontal arrow in the illustration of rice and wheat. In the illustration of *Nor-B2*, ISBP and genic markers that mapped to the five contigs are indicated by white and black boxes, respectively. The junction sequences of the ISBP markers are indicated in parentheses below the marker name. The uncharacterized genomic region within the contigs is represented by a dashed horizontal line.
