# Supplementary material for: A high-resolution physical map integrating an anchored chromosome with the BAC physical maps of wheat chromosome 6B
Source: BMC Genomics. 2015 Aug 12;16(1):595. doi: 10.1186/s12864-015-1803-y (PMC4534020; doi:10.1186/s12864-015-1803-y)
Supplement: Additional file 7: — Relationship of the gene positions between wheat chromosome 6B and the syntenic chromosomes of grass species. Dot plot of the best matches between genes on wheat chromosome 6B and the syntenic chromosomes: (A) 6B vs. rice chromosome 2 (Os02); (B) 6B vs. B. distachyon chromosome 3 (Bradi3); and (C) 6B vs. sorghum chromosome 4 (Sb04). The horizontal axis in the dot plots indicates the position of assigned genes on the 6B physical map, and the vertical axis indicates the physical positions of genes on each syntenic chromosome. (PDF 259 kb) [file 12864_2015_1803_MOESM7_ESM.pdf]

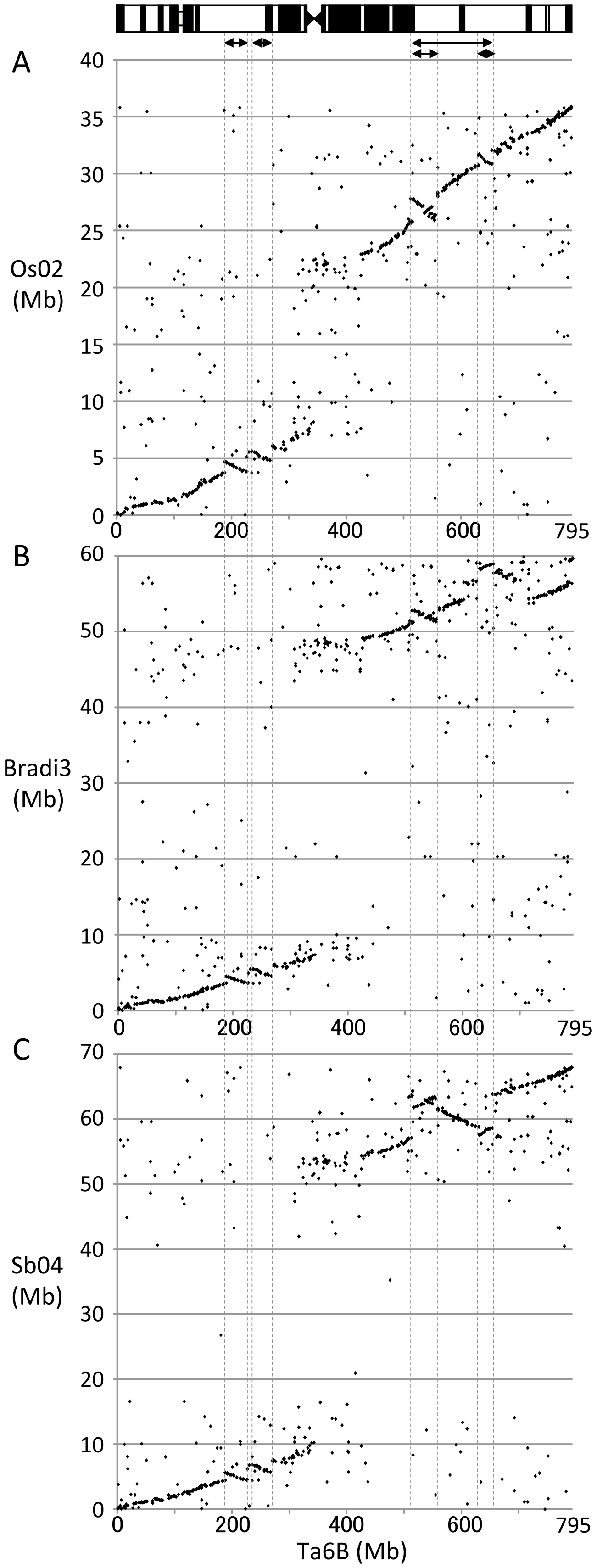

Additional file 7 Relationship of the gene positions between wheat chromosome 6B and the syntenic chromosomes of grass species.

Dot plot of the best matches between genes on wheat chromosome 6B and the syntenic chromosomes: (A) 6B vs. rice chromosome 2 (Os02); (B) 6B vs. *B. distachyon* chromosome 3 (Bradi3); and (C) 6B vs. sorghum chromosome 4 (Sb04). The horizontal axis in the dot plots indicates the position of assigned genes on the 6B physical map, and the vertical axis indicates the physical positions of genes on each syntenic chromosome.
